# Safety and tolerability of low dose oral minoxidil monotherapy in female pattern hair loss: A retrospective review with longitudinal ambulatory blood pressure monitoring

**DOI:** 10.1016/j.jdin.2023.08.002

**Published:** 2023-08-12

**Authors:** Reese Imhof, Beija Villalpando, Rochelle Torgerson

**Affiliations:** a Department of Dermatology, Mayo Clinic, Rochester, Minnesota; b Department of Medicine, Mayo Clinic, Rochester, Minnesota

**Keywords:** alopecia, androgenetic alopecia, female pattern hair loss, hair loss, low dose oral minoxidil, minoxidil, oral minoxidil

*To the Editor:*

Low dose oral minoxidil (LDOM) has been reported to be an effective and well-tolerated treatment in patients with various hair loss conditions.^1^ Previous studies have examined the cardiovascular effects and changes in the blood pressure (BP) in males on LDOM.^2^^,^^3^ A few studies have reported on female patients receiving LDOM as monotherapy for female pattern hair loss (FPHL). The objective of our study was to evaluate the effects of LDOM in female patients with FPHL.

We performed a retrospective review of adult female patients treated with LDOM (2.5 mg daily or less) for FPHL, who were evaluated by Mayo Clinic Dermatology in Rochester, Minnesota, over a 5-year period, through November 27, 2022. Patients were excluded if they were not solely diagnosed with FPHL, were receiving any other treatment, and did not have at least 4 months of posttreatment follow-up data with documented ambulatory vital signs.

A total of 25 patients with a mean age of 61 years (range: 28-77 years) met the inclusion criteria. The most common LDOM strength was 1.25 mg daily (13, 52%), followed by 2.5 mg daily (10, 40%), and 0.625 mg daily (2, 8%). Mean duration of the treatment was 6.2 months (range: 4-11 months). Ten patients (40%) who had previously tried topical minoxidil, discontinued receiving treatment due to local side effects or adherence difficulty. Additionally, 3 patients (12%) had previously tried other systemic therapies (finasteride and spironolactone), which were discontinued due to adverse side effects. Disease improvement and hair regrowth was noted in 9 patients (36%) who were being treated with LDOM, while unaltered disease progression was noted in 3 patients (12%). Five patients (20%) reported adverse side effects due to the LDOM treatment, including facial hypertrichosis (4, 16%) and fluid retention/lower limb edema (1, 4%). The one patient who developed edema discontinued the treatment. The patients who developed hypertrichosis did not discontinue LDOM. Figs 1 and 2 illustrate comparisons between patients’ baseline ambulatory BP and heart rate (HR) before initiating the LDOM treatment and their subsequent ambulatory BP and HR after at least 4 months of treatment. The baseline systolic BP (SBP) ranged from 107 to 161 mmHg, baseline diastolic BP (DBP) ranged from 58 to 88 mmHg, and baseline HR from 54 to 114 beats/min. The posttreatment SBP ranged from 102 to 152 mmHg, DBP ranged from 63 to 90 mmHg, and HR ranged from 56 to 105 beats/min. There was a mean change in SBP of −2.8 mmHg, in DBP of −1.4 mmHg, and in HR of +4.4 beats/min.

**Fig 1:**
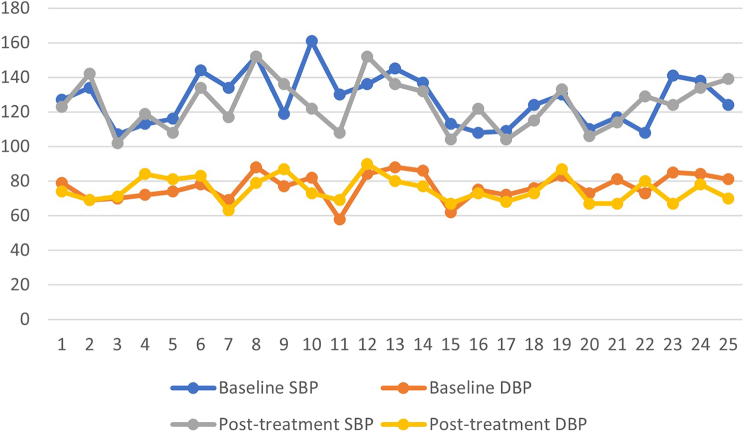
Baseline ambulatory systolic blood pressure (SBP) and diastolic blood pressure (DBP) measurements of patients before the initiation of low dose oral minoxidil (LDOM) compared to follow-up ambulatory SBP and DBP after at least 4 months of treatment with LDOM.

**Fig 2:**
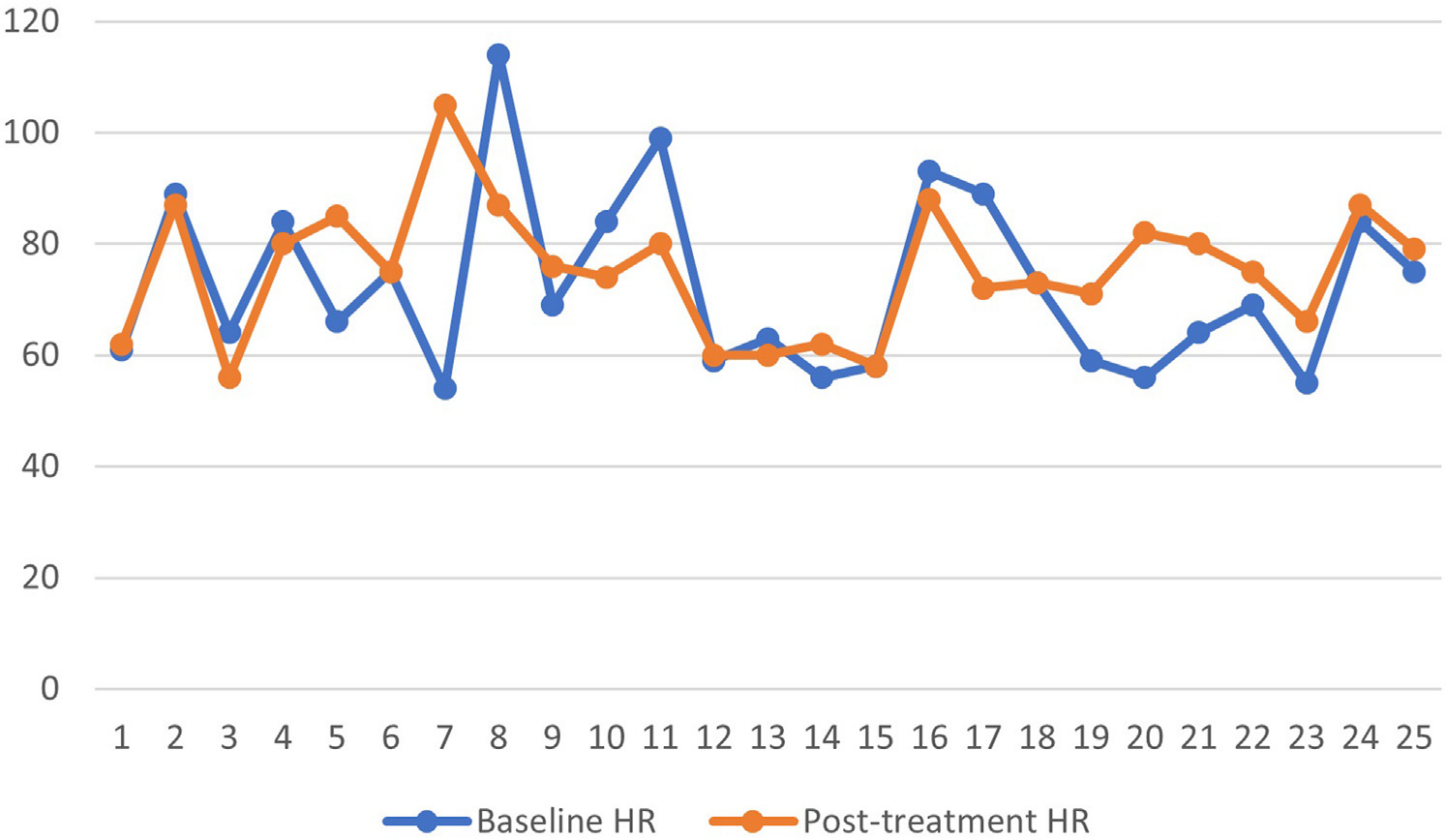
Baseline ambulatory heart rate (HR) measurements of patients before the initiation of low dose oral minoxidil (LDOM) compared to follow-up ambulatory HR after at least 4 months of treatment with LDOM.

In our study, there were minimal changes in SBP, DBP, and HR in female patients who were being treated for FPHL with LDOM monotherapy for at least 4 months. In an existing 24-week randomized clinical trial, no differences were reported in the mean BP variation between minoxidil 1 mg oral and minoxidil 5% topical solution for FPHL. However, in the oral minoxidil group, there was a mean HR increase of 6.5% without tachycardia.^4^ Participants in this trial who were on oral minoxidil had a mean age of 40.6 years and were subjected to a maximum daily dose of 1 mg.^4^ While our study’s findings are similar with respect to the BP and HR changes, the patients in our study were older (mean age: 61 years) and were subjected to higher doses of LDOM. Limitations to our study include the small sample size and lack of patients on concomitant antihypertensive medications.

## Conflicts of interest

None disclosed.
